# Correlations Between Depression Severity and Socioeconomic and Political Factors in Women over 50: A Longitudinal Study in Europe

**DOI:** 10.3390/healthcare14010042

**Published:** 2025-12-23

**Authors:** Lee Lusher, Samuel Giesser, David A. Groneberg, Stefanie Mache

**Affiliations:** 1 Institute of Occupational, Social and Environmental Medicine, Goethe University, Theodor-Stern-Kai 7, 60590 Frankfurt, Germany; inesleelusher@gmail.com (L.L.); samgiesser@gmail.com (S.G.); groneberg@med.uni-frankfurt.de (D.A.G.); 2 Institute for Occupational and Maritime Medicine (ZfAM), University Medical Center Hamburg-Eppendorf (UKE), Seewartenstraße 10, Haus 1, 20246 Hamburg, Germany

**Keywords:** depression, older women, mental health, SHARE study, EURO-D, socioeconomic factors, political factors, ageing population

## Abstract

**Background:** With ageing populations and increasing labour force participation among women over 50, their mental health and psychological well-being require attention. The multifactorial etiology of depression has been extensively studied at both the individual and societal levels. Longitudinal analyses exploring socioeconomic and political determinants and whether they influence depression severity across European countries are lacking. Objective: The objective of this study was to examine a possible correlation between socioeconomic and political factors with depression severity in women aged 50 and older in Europe and to what extent these possible correlations vary across countries. **Methods:** This longitudinal observational study was conducted using data from 47,426 women aged 50–89 years across 15 European countries, drawn from seven waves (2004–2015) of the Survey of Health, Ageing and Retirement in Europe (SHARE). Depression symptoms were measured by the validated European Depression Scale (EURO-D). The Andersen Model of Health Service Utilization was applied to contextualize twelve macro-level predictors of depression. These were organized into four overarching domains—health, education/employment/finance, equality, and security. Mean EURO-D scores were calculated with respect to age group and country. Correlations between predictors and depressive symptoms were assessed using Pearson’s and Adjusted Pearson’s correlation coefficients to determine the strength and rank of associations. **Results:** Significant correlations between predictor variables and depression were identified in nine countries, especially among women aged 80–89 years. Spain and Estonia showed strong predictors across several age groups. Eastern European countries exhibited the broadest range of significant correlations. Italy and France, despite high depression levels, revealed few significant predictors. Sweden, the Netherlands, and Switzerland had lower depression scores and demonstrated clearer correlations. Factors related to LGBTQ+ rights, perceived corruption, and peace indices emerged as influential. **Conclusions:** Country-specific historical, cultural, and sociopolitical factors appear to shape severity of depression in older women, with the strongest effects in the oldest age groups. Predictors of EURO-D scores varied by country and age group, with differences in explanatory power. The importance of predictors varied across age groups; listing them without context misrepresents the findings. The interplay between objective indicators and public perception, especially concerning minority rights and governance, highlights the need for culturally sensitive interventions. Future prevention efforts should incorporate these determinants to improve mental health across Europe.

## 1. Introduction

Depression is one of the most prevalent mental health disorders worldwide, contributing substantially to disability and economic burden—over €22 billion annually in Germany and more than $1 trillion globally [1,2]. Risk factors are multifactorial, spanning biological, psychological, and social domains, and include socioeconomic hardship, chronic illness, structural inequality, and political instability [3,4,5,6,7,8,9,10,11]. With rising life expectancy and declining birth rates, the proportion of older adults is increasing; by 2050, one-third of the population in OECD (Organisation for Economic Co-operation and Development) countries will be over 65 [12]. Depression peaks in later life—in 2019, depression affected 280 million people worldwide, with prevalence being the highest among women [13,14,15]. Women over 50 face distinct vulnerabilities arising from the intersecting effects of ageing, gender inequality, and shifting patterns of labour participation. These dynamics can heighten depression risk—through increased exposure to work-related stress and age- or gender-based discrimination or, conversely, through the loss of psychosocial and financial benefits when leaving employment—while caregiving responsibilities add further strain. Yet, the structural drivers of depression in this group remain insufficiently examined.

Previous studies, including ESEMeD, LASA, and SHARE (Survey of Health, Ageing and Retirement in Europe), reveal cross-national variation in depression prevalence and highlight the role of social isolation, widowhood, economic insecurity, and limited healthcare access [16,17,18,19,20,21,22,23,24,25,26]. The WHO’s 2022 (World Health Organisation) World Mental Health Report emphasizes that broader societal forces—such as inequality, governance, and conflict—shape population mental health [27]. However, most research has examined these determinants in isolation, and few studies have systematically compared their combined effects across countries.

This study addresses this gap by investigating the influence of socioeconomic and political factors and the severity of depression in women aged 50 years and older across 15 European countries. We chose to focus on this group because women in later life represent a high-risk population with distinct structural vulnerabilities—such as lower lifetime earnings, greater caregiving responsibilities, and higher rates of widowhood—making their risk profile and policy needs meaningfully different from those of men [26,28,29,30,31,32].

### Theoretical Framework

This study is grounded in the Andersen Model of Health Service Utilization, which conceptualizes health outcomes as the result of interactions among predisposing, enabling, and need factors [33]. Predisposing factors encompass demographic and social characteristics that shape vulnerability; enabling factors refer to structural and financial resources that facilitate or constrain access to care; and need factors capture both perceived and objective health needs.

We apply this framework to examine macro-level determinants of depression in older women across Europe. National characteristics such as health system capacity, economic security, social equality, and governance structures are conceptualized as contextual conditions that shape depressive symptomatology through the Andersen domains. This approach provides a theoretically coherent rationale for linking country-level socioeconomic and political environments to population-level mental health outcomes.

To enhance the construct validity of the macro-level predictors included in the analysis, we incorporated supplementary country-level plausibility indicators selected a priori. Selection followed four criteria:

(1) theoretical relevance to the Andersen model,
(2) empirical support from prior research,
(3) conceptual independence from the primary predictors to avoid circularity, and
(4) availability of harmonized cross-national time series.

These supplementary indicators span the domains of Health, Education/Employment/Finance, Equality, and Inner Security and are conceptually aligned with the predisposing, enabling, and need factors defined by the Andersen framework [33]. Their integration supports a comprehensive understanding of how macro-structural contexts shape depression risk in later life. A complete overview of all primary and supplementary indicators, including definitions, domains, and data sources, is presented in Table 1.

Based on this conceptual foundation, the study addresses the central research question:

“Are socioeconomic and political factors correlated with depression severity in women aged 50 to 89, and to what extent do these correlations vary across countries?”

By identifying structural determinants that differ between national contexts, the study aims to inform mental health policy development beyond the healthcare sector, highlighting the importance of coordinated social, economic, and political interventions.

## 2. Methods

### 2.1. Study Design and Data Source

We conducted a repeated cross-sectional, age-stratified study using individual-level data from the SHARE survey (2004–2015) combined with country-level macro indicators capturing health system capacity, economic security, social equality, and governance. This design enhances comparability across countries and years while avoiding the constraints of individual-level longitudinal modelling.

SHARE is a biennial panel study of individuals aged 50 and older across 28 European countries that collects detailed information on health, socioeconomic conditions, and social networks. To maintain representativeness, SHARE supplements its longitudinal sample with new probability-based recruits at each wave. For this analysis, we used data from Waves 1–6, corresponding to survey years 2004, 2006–2007, 2008–2009, 2010–2011, 2013, and 2015 [35,36,37,38,39,40,41].

SHARE data are collected in repeated ‘waves’, each corresponding to a specific cross-national survey period. For this analysis, we used Waves 1–7, corresponding to the following years: Wave 1—2004; Wave 2—2006–2007; Wave 3—2008–2009; Wave 4—2010–2011; Wave 5—2013; and Wave 6—2015 [35,36,37,38,39,40,41].

Data collection for each SHARE wave begins with a written invitation to potential participants, who receive a unique identification number upon consent. Trained interviewers then conduct face-to-face, in-home interviews at scheduled appointments, with telephone interviews used only in exceptional cases. To maintain representativeness over time, SHARE periodically recruits new participants using probability-based sampling from national population registers or similar sources.

We employed a repeated cross-sectional design stratified by age groups (50–59, 60–69, 70–79, 80–89 years). Some individuals appear in multiple waves, but no individual-level longitudinal modelling was conducted. This approach enabled comparable analyses across countries and waves while avoiding dependence among repeated observations of the same individuals. Age stratification ensured robust examination of population-level associations between macroeconomic and political predictors and depression severity, which was the primary aim of this study.

To ensure temporal and geographic alignment, all country-level predictors were matched to individual EURO-D scores for the same years and countries, with this matching performed separately by age group. This approach avoided averaging across years or countries.

Data from SHARE waves 1, 2, and 4 through 6 (2004–2015) were used. The final sample comprises 15 European countries: Austria, Belgium, Switzerland, Germany, Denmark, Spain, France, Greece (waves 1 and 2), Italy, the Netherlands, Sweden, the Czech Republic, Poland, Estonia, and Slovenia.

### 2.2. Sample Selection

Given our aim to investigate a high-risk group characterized by specific socioeconomic and political vulnerabilities, the study focused exclusively on women aged 50–89 years. This choice, rather than pursuing gender-comparative or intersectionality-oriented analyses, aligns with SHARE’s target population of individuals aged 50 and above. The final analytic sample consists 47,426 women aged 50–89 years from 15 European countries with complete EURO-D data.

### 2.3. Measurements

#### 2.3.1. Outcome Variable: Depression

The primary outcome variable is the EURO-D depression score, derived from SHARE’s “Mental Health” module. The items assess depressed mood, pessimism, suicidal ideation, guilt, sleep disturbance, irritability, loss of interest, lack of enjoyment, appetite changes, fatigue, concentration difficulties, and tearfulness. Each item is scored 0 (symptom absent) or 1 (symptom present), resulting in a total score ranging from 0 to 12, with higher scores indicating greater depressive symptom severity. It has been extensively validated for use in older European populations and allows for cross-country comparability of depressive symptomatology. A cut-off score of ≥3 is widely accepted to indicate clinically relevant depression, enabling the identification of individuals at increased risk for adverse health outcomes and mortality [42].

The EURO-D scale demonstrates good to very good internal consistency across diverse populations, with Cronbach’s alpha values ranging from 0.7 to 0.9, reflecting reliable measurement of depressive symptoms [43]. Factor analytic studies confirm a two-factor structure comprising two related dimensions: *Depression* (core mood symptoms) and *Affective Distress/Lack of Motivation* (anhedonia and psychomotor changes), with respective reliabilities of 0.83 and 0.79 [44]. Criterion validity of the EURO-D is well-established through strong correlations with other validated depression instruments, such as the CES-D and GDS, supporting its use in epidemiological research on ageing populations [44].

The continuous EURO-D score was used for analyses to capture variations in depressive symptom severity, while the established clinical cut-off facilitated interpretation of findings with respect to clinically relevant depression prevalence.

#### 2.3.2. Predictor Variables

The SHARE dataset provides robust individual-level predictors of depression (e.g., partnership status, health, life satisfaction). This study, however, focuses on macro-level societal and policy factors to capture structural determinants influencing mental health in ageing populations. Although these predictors are not specific to women aged 50–89, they reflect broad structural and societal conditions affecting the entire population, including older women. Our aim was to incorporate contextual factors beyond individual characteristics that shape mental health outcomes. While these indices are not psychometric tools, they are commonly used in public health research and supported by empirical and review evidence as valid measures of the social, economic, and political environments impacting population mental health [45,46,47]. Guided by this evidence, we selected twelve country-level variables (Table 1) that are well-established in the literature on social determinants of mental health in older adults, with specific attention to the vulnerabilities of women aged 50+ [32,48,49]. These variables were organized into four thematic domains:

(1) **Health system capacity and quality**—The Healthcare Access and Quality Index (HAQ) from the Institute for Health Metrics and Evaluation (IHME), the psychiatrist-to-population ratio per 100,000 inhabitants reported by the OECD, and health expenditure as a percentage of Gross Domestic Product (GDP_H_) from the OECD. These indicators capture population health outcomes, mental health workforce availability, and resource investment, all of which are linked to depressive outcomes and mediate socioeconomic disparities in late-life mental health [48].
(2) **Education, employment, and financial security**—The Educational Index (EI) from the United Nations (UN) and the unemployment rate (UR) from the OECD (using first-quarter data for temporal consistency), and Pension Purchasing Power Standard (PPS_Pension_), calculated as:
  $${PPS}_{Pension}=\frac{Pension}{{PPP}_{Country}}$$

- (PPP_Country_ = country-specific Purchasing Power Parity. Pension = Main Public Pension Income from SHARE “Employment and Pensions” module)
- These indicators allow for cross-national comparisons of old-age poverty and economic security, both consistently associated with depression risk in older women [49].

(3) **Equality and inclusion**—The Gender Inequality Index (GII) and the Gini Index (GI) from the United Nations (UN), and the Rainbow Europe Index (REI) from ILGA-Europe (International Lesbian, Gay, Bisexual, Trans and Intersex Association—Europe). These reflect gender disparities, overall income inequality, and LGBTQ+ rights/social inclusion, respectively. The Gini index does not capture absolute income levels but reflects the degree of income inequality within a country, serving as a proxy for structural socioeconomic disparities. Higher inequality and reduced inclusion are associated with elevated depression prevalence, particularly in groups experiencing cumulative disadvantage [32,50].
(4) **Governance and security**—The Global Peace Index (GPI) from the Institute for Economics and Peace, the Control of Corruption Index (CCI) from the World Bank, and the Immigration Ratio (IR) from the OECD. These capture political stability, governance quality, and societal safety, which influence population-level mental health; weaker governance contexts show higher rates of depression in vulnerable groups [46,51].

The ecological nature of these indicators means they cannot be interpreted as individual-level risk factors, and their use requires cautious interpretation.

We evaluated conceptual overlap between indicators to ensure distinct constructs were captured (Table 1). Redundancy between HAQ and psychiatrist density is low, as they measure different health system dimensions. HAQ and health expenditure show moderate overlap, since both relate to healthcare performance; however, expenditure reflects financial input, whereas HAQ captures system effectiveness and outcomes. Psychiatrist density and health expenditure are related due to shared personnel cost structures and interact with each other without overlapping. Economic indicators such as unemployment rate and PPS pension influence each other through shared macroeconomic dynamics, while education remains distinct from the other indices. Social inequality measures show partial (GII–Gini) or minimal (REI–Gini) overlap, with REI complementary to GII. Whereas the Gini index reflects overall wealth distribution in a society, the GII captures women’s empowerment within a society. Governance measures (GPI and CCI) overlap moderately, as both are shaped by institutional strength; however, the GPI assesses peace and security, while the CCI reflects integrity of governance. Immigration ratio is conceptually independent from the other indices.

### 2.4. Selection of Macro-Level Indicators

The analysis was guided by the Andersen Model of Health Service Utilization, which conceptualizes health outcomes as the result of predisposing, enabling, and need factors [33]. This framework was used to classify all macro-level predictors of depression. Predisposing factors capture demographic and structural characteristics shaping vulnerability; enabling factors denote institutional, financial, or structural resources that facilitate or hinder access to care; and need factors reflect objective or perceived health needs, operationalized in this study using the EURO-D depression score and additional mental-health–related indicators.

To ensure conceptual robustness, primary macro-level predictors were complemented by supplementary country-level plausibility indicators. These were selected a priori according to four criteria: (1) conceptual alignment with the Andersen framework, (2) empirical support in the literature, (3) independence from primary predictors to prevent circular reasoning, and (4) availability of harmonized, cross-national time series. All indicators relate to one of four thematic domains—Health, Education/Employment/Finance, Equality, and Inner Security—and were assigned to the Andersen categories accordingly.

Within the **Health** domain, Out-of-Pocket Health Expenditure (enabling factor) captures financial barriers to accessing care and has been linked to increased depression risk, particularly in the context of high health expenditures ([52]; WHO annual data since 2000). The Female Suicide Rate (need factor) provides an independent indicator of mental health burden and reflects interactions between healthcare access, social support, and health system functioning (WHO annual data since 2000). Female Life Expectancy (predisposing factor) serves as a broad indicator of systemic health inequalities and has been collected annually by the WHO since 1960.

In the domain of **Education, Employment, and Finance**, the proportion of women aged ≥50 with tertiary education (predisposing factor) reflects cognitive reserve and labour market opportunities, with annual UNECE data available since 2005 [53]. The proportion of women in STEM fields (predisposing factor) captures occupational gender segregation and structural barriers in education-to-employment pathways, influencing depression risk; World Bank annual data are available since 1998 [54]. The Risk of Poverty in Women aged ≥65 (enabling factor) reflects financial insecurity in later life, based on Eurostat data collected annually since 2015. The Index of Horizontal Segregation (predisposing factor) quantifies concentration in lower-paid or less secure occupational sectors and was calculated by Meulders et al. (2010) [34,55].

The **Equality** domain includes the Global Gender Gap Index (predisposing factor), a composite measure of gender equality across multiple societal dimensions, available annually from the World Economic Forum since 2006 [56]. The Gender Wage Gap (predisposing factor), collected by Eurostat since 2006 [29,57], and the Gender Pension Gap (predisposing factor), collected by Eurostat since 2010 [30], reflect cumulative disparities in earnings and retirement income; the latter is conceptually linked to overall asset inequality as measured by the Gini Index [58]. Qualitative contextual information on the historical and policy environment—including past discriminatory laws and the timing of equality legislation—was used to further situate structural determinants relevant to gender and LGBTQ+ rights.

Indicators in the **Inner Security** domain capture structural conditions associated with public safety and governance. The Robbery Rate per 100,000 population (predisposing factor) reflects crime-related insecurity and aligns conceptually with the Global Peace Index (annual data available from The Global Economy since 2009) [59]. The Corruption Perceptions Index (predisposing factor) assesses governance quality and institutional trust, collected annually by Transparency International since 1995 [60,61]. The Prevalence of Interpersonal Violence (need factor) represents gender-specific vulnerabilities associated with weak governance and has been collected sporadically by the OECD since 2000 [31]. Interpersonal violence and corruption indicators are conceptually related to broader processes such as immigration, social cohesion, and institutional responses to violence [62,63].

### 2.5. Missing Data

Wave 3 was excluded due to missing depression measures (EURO-D), and Greek data from Wave 6 were omitted because of the eight-year gap since Wave 2, which limited longitudinal comparability. Several countries either lacked consistent EURO-D measurement or joined SHARE after the observation period (e.g., Bulgaria, Ireland, Hungary, Portugal, Croatia, Luxembourg, Cyprus, Latvia, Lithuania, Malta, Slovakia, Romania, Finland, UK), precluding their inclusion. Israel was excluded due to its non-European context.

From the initial SHARE dataset of 107,554 participants, 3.5% were outside the target age range (< 50 or >90 years), 44.5% were men, and 8.7% had incomplete EURO-D depression scale data. Missing data were mainly due to non-response on EURO-D items or the availability of only a single EURO-D measurement. On average, 2–3 % of EURO-D values were missing, with only minor variation across countries and survey waves. Attrition increased in later waves, as expected in ageing cohorts.

*Outcome variable (EURO-D):* No imputation or interpolation was applied to EURO-D scores in order to preserve the validity of the depression outcome. Participants with missing values were excluded from analyses for the relevant variables. Missing values arose either from non-response to individual EURO-D items or from respondents providing only a single EURO-D measurement. Across the waves included in this study, the proportion of missing EURO-D values ranged from 2.7% (Wave 1) to 5.1% (Wave 6).

*Predictor variables:* Similarly, no imputation or interpolation was performed for Main Public Pension Income. Participants with missing values were excluded from analyses for the relevant variables. Missing values resulted either from non-response or from respondents providing only a single measurement. The proportion of missing Main Public Pension Income data across waves ranged from 4.3% (Wave 6) to 6.5% (Wave 1). All other predictor variables were complete, as they were derived from publicly available sources or indices.

Baseline comparisons indicated that excluded participants were, on average, older, less educated, and had higher initial EURO-D scores than those retained in the analysis. These differences suggest that depression prevalence in the analytic sample may be slightly underestimated.

### 2.6. Data Preparation

Participants were stratified into four predefined age groups (50–59, 60–69, 70–79, 80–89 years) based on established categorizations in ageing research and public health policy, consistent with the OECD’s framework for studying ageing populations to enable age-specific analyses of depression and its predictors [64]. These age-groups reflect meaningful life stages with distinct social roles, health, and vulnerabilities, enabling a nuanced analysis of socioeconomic and political factors relating to depression across later adulthood.

For each participant, average EURO-D scores, and socioeconomic indicators were calculated over the study period to provide summary measures of average exposure and depressive symptomatology. We characterized variability and statistical precision using standard deviations and 95% confidence intervals. Summary statistics were stratified by age group and country to capture sample heterogeneity.

### 2.7. Plausibility Assessment of Cross-Country Differences in EURO-D Scores

To evaluate the plausibility of country-level differences in EURO-D scores, a two-step approach was employed. A theoretical expectation was first established by examining whether a plausible, content-based relationship existed between the country-level plausibility criteria (Table 1) and the corresponding EURO-D scores. Each plausibility criterion was compared across all participating countries to assess whether cross-country variation in these indicators could plausibly relate to the EURO-D score of the respective country. In the second step, empirical associations between each predictor and the country-specific EURO-D scores were quantified using appropriate statistical methods. The empirical findings were then systematically compared with the previously established theoretical expectations. Consistency between theoretical predictions and empirical results was interpreted as supporting the plausibility of the observed cross-country differences in EURO-D scores. Any discrepancies between expected and observed associations were identified and discussed as potential limitations or indicators of context-specific modifying factors.

### 2.8. Statistical Analysis

**Figure 1:**
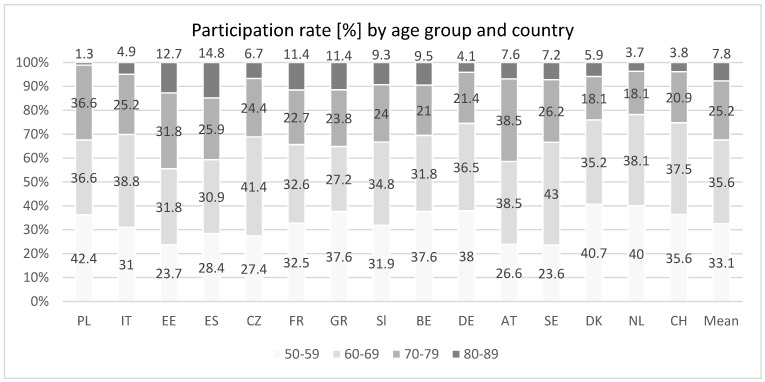
Participation rates (%) by age group and country.

**Figure 2:**
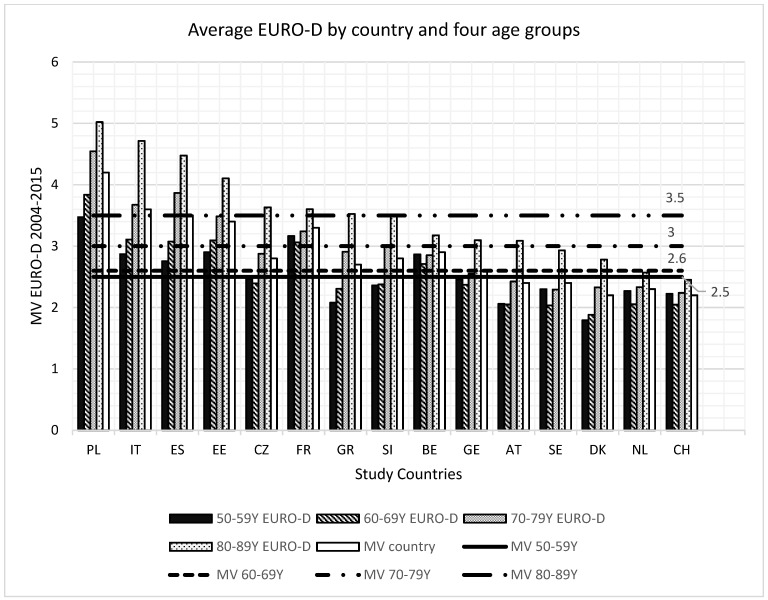
EURO-D mean values by country and four age groups; MV = Mean Value.

We provide an overview of the selected cohort within the SHARE study by providing a tabular format (Table 2) of the number of study participants by waves and country as well as the number of study participants by age across each included country in the study (Figure 1). For further information we provide the average EURO-D scores across the different age-groups per country and examine which countries and age-groups have EURO-D scores exceeding the clinically significant EURO-D threshold of three (Figure 2). 

The publication aims to examine and quantify the influence of socioeconomic factors on population-level EURO-D scores. To this end, we construct linear models to assess the predictive power of socioeconomic variables on the EURO-D score.

For each participant and each corresponding year, each of the predictor variables were paired with the respective EURO-D score, resulting in a data cloud in which the correlation between the two variables was examined. Each participant could contribute multiple observations according to the number of waves in which they participated. The association was quantified using Pearson’s correlation coefficient. Model input variables were then selected based on those correlations with EURO-D scores to reduce model dimensionality and focus on the most promising predictors. To select meaningful associations, we applied dual criteria based on both effect size and statistical significance. The strength of an association was determined by the absolute value of the Pearson correlation coefficient (r), with larger |r| indicating a stronger linear relationship. Only countries meeting both criteria (existence of an r > 0.09 and *p* < 0.05) were included in further analyses. The r > 0.09 threshold was empirically chosen to ensure that the countries retained for further analysis consistently showed stronger associations than those excluded within the same age group.

For each country and age group, we then fit a linear regression model using backward elimination based on *p*-values, removing variables until all remaining predictors have a variance inflation factor (VIF) below five.

Finally, we record the adjusted Pearson correlation coefficient for each model, allowing for comparison of the explanatory power of socioeconomic factors across different countries and age groups, independent of the number of predictors included.

These adjusted Pearson coefficients quantified the strength of association between each predictor and EURO-D scores and were ranked by magnitude within each country and age group. We recorded the rank-strength of the individual included predictors. The model results and ranked predictive power per country and age group is recorded in Table 3.

All statistical analyses were performed using R (version 4.4.2) and Microsoft Excel 2016. A significance level of α = 0.05 was applied throughout.

### 2.9. Ethics and Data Availability

SHARE received ethical approval from the University of Mannheim Ethics Committee (waves 1–3) and the Max Planck Society Ethics Council (waves 4–9). National implementations were approved by local ethics committees [65]. The SHARE dataset is publicly accessible, and the data used in this study were retrieved and filtered on 1 June 2023.

## 3. Results

The number of study participants varied considerably across survey waves and age groups (Table 2). The majority of respondents were in the 50–59 and 60–69 age brackets, with participant numbers declining progressively in older age groups, and the smallest sample observed in the 80–89 group. Wave 5 recorded the highest number of participants (*n* = 14,186), while wave 1 had the lowest (*n* = 5243).

The proportion of participants aged 80–89 was consistently lower than younger groups across all countries (Figure 1). Poland had the largest share of participants aged 50–59 (42.4%) and the smallest in the oldest group (1.3%). Estonia and Spain showed the highest relative participation among women aged 80–89 (12.7% and 14.8%, respectively). Austria had the greatest representation in the 70–79 age group (38.5%), and Sweden led in the 60–69 group.

Mean EURO-D scores revealed that only Poland and France consistently exceeded the clinical depression threshold of ≥3 across all age groups (Figure 2). Eastern European countries—Poland, Estonia, the Czech Republic, and Slovenia—reported the highest mean EURO-D scores, ranging from 2.8 (Czech Republic and Slovenia) to 4.2 (Poland). In contrast, Central and Northern European countries such as Switzerland and Belgium exhibited the lowest mean values (2.2 and 2.9, respectively).

Pearson correlation analyses demonstrated notable regional variation in the strength and significance of associations between depressive symptoms and socioeconomic or political predictors (Table 3). Significant associations were most pronounced in women aged 80–89 years, except in Estonia and Spain, where meaningful correlations were observed in the 60–89 and 60–79 age ranges, respectively.

Despite relatively shorter observation periods (~5 years), Poland, the Czech Republic, and Estonia showed the highest adjusted correlation coefficients (r = 0.67–0.81; *p* < 0.044) among the oldest age group (Table 3). In Estonia, clinically relevant EURO-D scores (>3) were observed beginning at age 70. Spain, with the longest follow-up (11 years) and across two age groups (60–79), displayed an adjusted correlation of r = 0.65 and associations with five to seven predictors depending on age.

Conversely, Greece exhibited the lowest adjusted correlation (r = 0.32) despite a high mean EURO-D score of 3.5 and a brief observation period (3 years). France and Italy lacked statistically significant predictors despite consistently elevated depression scores above the clinical cutoff in all age groups.

Countries with longer observation periods—Switzerland (11 years), the Netherlands (9 years), and Sweden (11 years)—showed low to moderate correlation coefficients (r = 0.54–0.58) and were associated with three significant predictors each; these countries also reported mean EURO-D scores below the clinical threshold (EURO-D = 2.5–2.9).

The prevalence and explanatory power of key predictors varied markedly. Italy and France demonstrated elevated depression levels without significant correlates, whereas Poland and Spain exhibited age-specific associations with multiple predictors despite varying depression prevalence. Belgium, Germany, and Austria showed clinically relevant EURO-D scores in the 70–79 age group but lacked significant predictor associations (Figure 2).

## 4. Discussion

### 4.1. Strengths and Limitations

This study primarily aims to illustrate a conceptual framework linking macro-level socioeconomic and political factors to depression severity in older women, based on longitudinal data from 2004 to 2015. These findings should therefore be interpreted within their historical context. Whether the identified associations remain stable today, or whether new determinants have emerged, requires updated post-2015 data. While our results demonstrate which domains were relevant during the observation period, they do not claim to represent the current mental health landscape in Europe. Any contemporary relevance of individual predictors—such as changes in economic conditions, healthcare availability, political stability, or minority rights—cannot be inferred from the present analysis. Future research is needed to assess how ongoing demographic, sociopolitical, and economic changes influence depression risks in 2025 and beyond.

This eleven-year study examines depression in women aged 50 and older, drawing on a large and diverse sample of 47,426 participants across 15 European countries. The substantial sample size enhances statistical power and supports the generalizability of findings across different national contexts (Table 2). Moreover, the extended observation period allows for the analysis of both short- and long-term trends in depressive symptoms. The use of the harmonized EURO-D scale ensures consistency in the measurement of depressive symptoms across countries and waves.

However, several methodological limitations must be acknowledged.

First, the cross-national comparability of EURO-D assessments may be influenced by seasonal and diurnal variation in survey administration, potentially introducing measurement bias. Second, the alignment between the timing of predictor variables and EURO-D assessments presents challenges, as many predictors reflect broader societal trends rather than immediate, individual-level exposures or behaviours. This mismatch may attenuate the precision of the observed associations.

The EURO-D scale, although widely validated and suitable for cross-national research, captures depressive symptoms rather than clinical diagnoses. The measure may be sensitive to cultural differences in symptom reporting, which could affect cross-country comparisons.

A further methodological limitation arises from the difference in measurement level between the predictors and the outcome. The macro-level indicators used in this study—such as national economic indices, equality measures, and governance indicators—are aggregated, country-level constructs. In contrast, the EURO-D score is an individual-level measure of depressive symptoms. Because the levels of analysis do not match, macro-level variables cannot be interpreted as direct determinants of an individual’s depression severity. Any correlation identified in this study therefore reflects an ecological association at the population level rather than an effect on the individual. Consequently, the results should not be interpreted as implying that changes in a national indicator—such as GDP, corruption perception, or minority rights—would directly alter a person’s EURO-D score.

Notably, two of the twelve macro-level predictors—the Rainbow Equality Index (REI) and the Global Peace Index (GPI)—began data collection after the start of the SHARE study, in 2009 and 2007, respectively, whereas SHARE data collection commenced in 2004. This temporal mismatch somewhat limits the interpretability and strength of conclusions related to these predictors, as earlier trends or long-term associations may be underrepresented. The asynchronous availability of these predictors introduces heterogeneity in the temporal coverage of country-level variables, which may affect comparability of effects over time and potentially bias estimates. Therefore, findings related to REI and GPI should be interpreted with caution, and future studies would benefit from fully synchronized longitudinal macro-level data.

Additionally, in countries with shorter observation periods, the data may disproportionately reflect acute political or economic crises rather than sustained patterns, thereby limiting causal inference. Variation in participation rates across countries, age groups, and survey waves further complicates interpretation (Figure 1). For example, participants aged 80 and older, differences in life expectancy led to uneven representation, ranging from 14.8% in Spain to just 1.3% in Poland. Interestingly, Estonia achieved a relatively high participation rate of 12.7% in this age group despite its lower average life expectancy, possibly due to effective outreach or sampling strategies [66].

The analysis relies on country-level averages, which may obscure within-country heterogeneity. Regional disparities, urban–rural differences, and subgroup variations could not be addressed with the available data. Greece and Poland engaged a high proportion of women aged 50–59 despite relatively short observation windows (three to four years), while Estonia and Sweden reported lower participation in this age group despite longer study durations (four and eleven years, respectively). These discrepancies may reflect challenges in recruiting working-age women, particularly in rural or less urbanized regions.

Further limitations include the absence of sex-specific or age-specific macro indicators, which may dilute the precision of contextual effects for women aged 50–89. The ecological nature of these country-level variables restricts direct individual-level inference, necessitating careful interpretation.

The study is correlational in nature. Macro-level indicators were analyzed in relation to mean depression scores. While the findings highlight potential contextual influences on depressive symptoms, the observational design precludes conclusions about directionality or mechanisms. In addition, some predictors represent perceptions (e.g., perceived corruption), while others reflect objective indicators, which may influence their comparability and interpretation.

Although a broad set of socioeconomic and political variables was examined, the selection is not exhaustive. Other contextual factors—such as migration patterns, family structure, social support networks, or healthcare system reforms—may also play important roles but were beyond the scope of the current study.

Although this study leverages longitudinal data, analyses used a repeated cross-sectional design stratified by age group, rather than individual-level longitudinal modelling. Consequently, causal inferences about within-person changes over time cannot be made. The repeated participation of some individuals across waves could introduce data dependencies; however, the age stratification and lack of individual longitudinal analyses substantially mitigate this issue. Future studies employing longitudinal modelling to capture individual patterns over time would provide complementary insights into the macroeconomic and political determinants of depressive symptoms.

While this study benefits from a large, multi-country sample and a longitudinal design, limitations related to temporal alignment of data, potential overrepresentation of acute events, and uneven participation—particularly among older and working-age women—should be carefully considered when interpreting the findings. Given that all interviews were conducted face-to-face at times agreed with participants and over extended multi-month fieldwork periods, systematic bias related to the time of day is unlikely, and any seasonal effects would likely be randomly distributed across countries and waves.

### 4.2. Eastern Europe

Across Eastern Europe (Poland, Czechia, Estonia, Slovenia), depression prevalence among women aged ≥50 was consistently higher than in Southern and Central/Northern Europe (Figure 2). Associations between socio-structural predictors and EURO-D scores were statistically significant across all age groups, with the strongest correlations observed among women aged 80–89. In this subgroup, adjusted Pearson coefficients ranged from 0.67 to 0.81 in Poland, Czechia, and Estonia, whereas Slovenia showed a notably weaker association (r = 0.35) (Table 3). These correlations exceeded those observed in Southern Europe (Greece: r = 0.32; Spain: r = 0.65) and Central/Northern Europe (Sweden: r = 0.54; Switzerland: r = 0.54; Netherlands: r = 0.58), despite a shorter average follow-up period in Eastern Europe (5 vs. 10 years), underscoring the robustness of the associations.

Although **Poland** showed the highest overall EURO-D scores, significant predictor associations emerged only among women aged 80–89. This selective pattern may indicate reduced variability in predictor age distributions or unmeasured contextual influences among women aged 50–79. In Poland, the most influential predictors were gender inequality (GII), public safety (GPI), limited healthcare access (HAQ), corruption (CCI), discrimination against LGBTQ+ individuals (REI), and restrictive attitudes toward immigration (IR) (Table 3). These findings aligned with plausibility criteria: Poland ranked among the lowest in the Global Gender Gap Index [67], and women’s safety was evaluated as only moderate [68]. Public attitudes toward violence remained concerning; in 2019, 10% of respondents considered intimate partner violence acceptable, and 11–13% of men and 6–7% of women denied the existence of marital rape [68,69]. Economic barriers to healthcare were substantial, with 20% of households spending >10% of income on medical costs—disproportionately affecting older women, whose life expectancy was 2.1 years below the EU average [66,70]. Additional structural challenges included high perceived corruption (low CCI scores [71]) and persistent LGBTQ+ discrimination (e.g., limited protections for LGBTQ+ teachers [72]). Despite low immigration levels, exclusionary political discourse remained prevalent [73,74,75,76].

In **Czechia**, the strongest predictors of depression were GDP per capita in purchasing power standards (GDP_H_), unemployment (UR), and gender inequality (GII), followed by the Education Index (EI) and Gini coefficient (GI) (Table 3). These associations were consistent with contextual data: GDP_H_ lagged 2.6 percentage points behind the EU average of 9.6% [77], and although women’s unemployment rates matched the EU average, their poverty rates were twice as high as men’s [78]. Vulnerable groups included older, single, unemployed women with low educational attainment [79,80]. Czechia ranked low in the Global Gender Gap Index [54,67], and women carried disproportionate unpaid care duties [81,82]. Although female STEM participation met the study average (32%), representation in ICT was the lowest in the EU (9.9% vs. 16.1%) [83]. The IP-Index placed Czechia second highest at 28.5% (EU average: 25.2%) [84].

In **Estonia**, key predictors across ages 60–89 included healthcare access (HAQ), psychiatrists per 100,000 inhabitants (Psy/I), unemployment (UR), and immigration (IQ). Among women aged 80–89, pension purchasing power (PPSPension) became an additional major factor, while corruption control (CCI) and income inequality (GI) were downgraded (Table 3). Female life expectancy was 2.4 years below the EU average [66,85]. Psychiatric inpatient capacity declined from 186 to 53 beds per 100,000 residents over 14 years [86,87], and a 2023 report described mental healthcare as fragmented, underfunded, and hard to navigate [87]. Following the 2008 crisis, female unemployment averaged 14.1% (EU: 10.2%) [88]. Despite the lowest immigration rate in the sample (0.15%) [74,89], one-third of older adults expressed negative migration attitudes [90], potentially reflecting the region’s high elderly poverty rate (39%), among the highest in Europe [91,92]. Many women worked beyond retirement age, yet inequality in assets, in income and in wage gapspersisted (Gini: 33 vs. EU: 31; wage gap: 28%) [93]. Corruption was a particularly strong predictor among women >80, especially in healthcare and urban planning [94]. In this age group, elderly poverty ranked highest, followed by corruption and income inequality; these associations weakened with age, potentially due to the rising importance of pension purchasing power.

In **Slovenia**, education (EI) was the primary predictor of depression (Table 3). University graduation rates among women >50 exceeded the study average by 2% (46%) [95]. Wage growth for highly educated women surpassed OECD averages, but female employment remained 10 percentage points below male employment and increased only marginally over time [96], reflecting persistent gendered labour disparities.

While these associations do not establish causality, the findings suggest that structural conditions exert sustained influence across the life course, particularly in Estonia, where three consecutive age groups showed strong associations. Gender inequality, occupational segregation, and discriminatory attitudes in Poland and Czechia contribute to long-term social and psychological vulnerabilities. Limited healthcare access, high out-of-pocket expenditures, declining psychiatric capacity, and systemic corruption in Poland and Estonia constrain access to appropriate care. Poverty, unemployment, reduced pension purchasing power, perceived corruption, and concerns about immigration intensify needs among women aged ≥80, amplifying depression risk in the most vulnerable segments of society.

### 4.3. Southern Europe

In Southern Europe (Spain, Italy, France, and Greece), depression patterns among older women differ markedly from those in Eastern Europe. Age discrimination and traditional gender roles may increase vulnerability in this population [97,98,99]. Suicide rates reflect these differences: women in France have suicide rates approximately three times higher than those in Italy, mirroring elevated EURO-D scores among women aged ≥60 [100]. Mental illness stigma remains particularly pronounced in France, whereas stronger social integration in Italy may serve as a protective factor [101,102].

Unlike most Eastern European countries—Poland being an exception—Southern European countries showed few statistically significant predictors, despite clinically relevant EURO-D scores. In Spain, significant associations emerged in women aged 60–79, including healthcare indicators (HAQ for ages 60–79; Psy/I for ages 60–69), unemployment (UR), discrimination indices (REI for ages 60–79), asset inequality (GI for ages 60–69), corruption (CCI), and perceptions of safety (GPI). Among women aged 80–89, however, no predictors reached statistical significance, despite comparatively high mean scores (mean EURO-D 4.5). Italy and France similarly exhibited clinically relevant depression scores across all age groups without identifiable significant predictors, suggesting either influences beyond the measured variables or limited variability of predictor trajectories over time.

The **Spanish** findings align with documented socioeconomic pressures. Following the 2008 financial crisis, severe healthcare budget cuts led to extended waiting times (50–160 days), increased mortality, and excess winter deaths, disproportionately affecting older adults [98,99,103,104,105,106,107]. These effects correspond to the age groups represented in this study (60–79 years), whereas those aged ≥80 are not included in the underlying time series. Although elderly poverty in Spain remains below the EU average (13% versus 16%), the country experienced one of the highest unemployment rates in Europe during and after the crisis—reaching 25% and affecting even adults over 70 years of age who were still able to work [88,108,109].

Additional Spanish predictors relate to the REI, reflecting the legacy of LGBTQ+ persecution under the Franco regime. Institutions for the “re-education” of homosexual individuals used coercive measures such as electroshock and aversion therapy [110]. These historical traumas continue to affect the mental health of older LGBTQ+ adults, although recent public discourse on recognition and compensation may provide partial protective effects [110,111].

Exposure to violence and social unrest (GPI) may also contribute to psychological vulnerability. Spain records robbery rates above the EU median, and the long-term repercussions of the Basque conflict remain evident; between 2006 and 2009, 21 ETA attacks caused seven deaths and more than 200 injuries [112,113,114]. While actual corruption levels are below the OECD average, perceived corruption—especially in healthcare—is notably high [61], with 23% of respondents reporting informal payments or distrust in medical settings [115,116,117].

Among women aged 60–69, psychiatrist density (Psy/I) and inequality in assets (GI) were strongly associated with depression severity. For women aged 70–79, unemployment (UR) emerged as the strongest predictor, reflecting barriers to labour market re-entry among older adults. Similarly to Estonia, the presence of multiple overlapping predictors across age groups suggests complex mechanisms contributing to depression risk in older women.

In **Greece**, predictors demonstrated the weakest explanatory power in the entire study (r = 0.32), likely due to the short observation window of only three years. The study concluded just as the global financial crisis began, limiting the ability to examine long-term effects. During this period, substantial healthcare funding and mental health service cuts were implemented [118]. Psychiatrist availability (Psy/I) was the only significant predictor in the oldest age group, whereas HAQ did not reach significance (Table 3). Before the crisis, Greece reported suicide rates approximately 4.5 times lower than the EU average [100], which may reflect strong sociocultural protective factors or possible underreporting. After the austerity measures, suicide rates rose noticeably [100]. Across all examined predictors, the theoretically anticipated relationships with country-level EURO-D scores were consistently supported by the statistical analyses, with the sole exception of the Gender Inequality Index (GII). While the health and education subcomponents of the GII reflected near gender parity in most participating countries, marked inequalities remained in 2015 in the dimensions of political empowerment and women’s access to senior economic leadership roles [56].

In both Spain and Greece, structural, historical, and socioeconomic conditions interact with healthcare system constraints to shape perceived and actual mental health needs. In Spain, the co-occurrence of several predictors across age groups suggests intertwined mechanisms linking limited enabling resources to elevated need. In Greece, the short observation period and crisis-related austerity illustrate how temporal and policy factors may modulate these associations.

### 4.4. Central and Northern Europe

Central and Northern Europe is represented by Sweden, the Netherlands, and Switzerland (Table 3). In the Netherlands and Switzerland, EURO-D scores consistently remain below the threshold for clinically relevant depression; Denmark similarly shows low scores and lacks significant predictors. In contrast, Belgium, Germany, and Austria show EURO-D scores above the clinical threshold but without statistically significant predictors (Figure 2, Table 3). As in Southern Europe, this suggests that the selection of predictors for Central and Northern Europe may require reassessment.

Despite comparatively low EURO-D values, Sweden, the Netherlands, and Switzerland display statistically significant predictors in the oldest age group (Figure 2, Table 3). This pattern likely reflects well-performing healthcare systems: these countries rank among the top in the European Health Consumer Index (EHCI), surpassing Southern Europe [119]. Emphasis on preventive care and early intervention may reduce overall depression prevalence, allowing smaller variations in predictors to reach significance. Cultural factors may also contribute, as stigma toward mental illness is generally lower in Central and Northern Europe than in Eastern and Southern Europe [97,102], facilitating earlier help-seeking and treatment.

In the **Netherlands**, key predictors include safety and violence (GPI), immigration (IQ), and economic inequality (Gini, GI). During the study period, the country experienced several major criminal and terrorist incidents, including the assassinations of Pim Fortuyn (2002) and Theo van Gogh (2004) and a terrorist attack on King’s Day (2009) [120,121]. By 2005, nearly 30% of citizens reported being victims of crime [122], with robbery rates around 70 times higher than the median of study countries [112]. Domestic violence was the most common form of violence, affecting 40% of the population—85% of victims being women [123]. The average immigration rate was 0.6%, slightly below the EU average of 0.7% [74,89]. Crime statistics showed significant ethnic disparities: Northeast Asians had the lowest crime rate (0.21 relative to Dutch citizens), while immigrants from Islamic countries such as the Dutch Antilles had the highest (3.81) [124]. The violent attacks triggered retaliatory acts against religious institutions [124], and Deputy Prime Minister Gerrit Zalm declared a “war on terrorism” [125,126]. By 2012, asset and income inequality had risen sharply: the Gini coefficient increased by 14%, and the wealth gap widened by 23% compared with the 1980s [93,127]. Socially vulnerable groups—including single parents, individuals with lower education, and low-income earners—were disproportionately affected [127]. Rising inequality coincided with increased xenophobia and crime rates, deepening social divides [127,128].

In **Switzerland**, acceptance of diversity lagged behind the EU average, as illustrated by the nationwide minaret ban [129]. Rapp (2020) showed that tolerance toward minorities closely depends on perceived threats [130]. Accordingly, the LGBTQ+ Rights and Equality Index (REI) was the strongest predictor in Switzerland (Table 3). Conversion therapy remains legally unresolved, with parliamentary bodies opting for further study; an estimated 14,000 LGBTQ+ individuals have been exposed to such practices [131,132,133]. Healthcare access (HAQ) was the second most relevant predictor and is among the highest in Europe, although disparities persist in elderly rehabilitation, psychiatric and long-term care, and in specific medical services. Considerable regional variation exists, with superior outpatient care in urban areas and the highest hospital bed capacities in border regions [134]. Crime rates remained consistently low during the study period [135]; nonetheless, a 2022 study by Baier et al. identified a widening gap between decreasing objective crime rates and heightened public fear of crime, likely influenced by sensationalized media narratives [136].

A complete assessment of depression predictors in Central and Northern Europe is constrained by the absence of representative data for Austria, Belgium, Denmark, and Germany—four of seven countries in this region.

In **Sweden** the key predictors number of psychiatrists per inhabitants (Psy/I), immigration (IQ), and education index (EI). The Immigration Quotient emerged as the strongest significant predictor of country-level EURO-D scores. During the observation period, Sweden’s annual immigration rate averaged 0.87% of its population—0.24 percentage points above the mean of participating countries—and rose from 0.53% to 1.16%, while the median across countries declined [74,89]. Although the Global Peace Index itself was not statistically significant, Sweden exhibits one of the highest reported rates of intimate partner violence (IPV) against women under 50 years among participating countries [31]. The GPI does not capture gender-based violence, contributing to its underrepresentation in composite peace metrics. The Education Index ranked second among significant predictors. Swedish women aged >50 years display the second-highest tertiary education attainment across all countries and genders, surpassed only by Estonia [95]. Despite this, only 59% of university-educated women work full-time (vs. 83% of men), and the financial return on higher education is among the lowest in the OECD, with the wage premium for a master’s degree being the smallest among Nordic countries [137].

In the Netherlands, Switzerland and Sweden, structural conditions—including exposure to violence, historical discrimination, and economic inequality—interact with system-level factors such as immigration issues, healthcare access and regional service disparities. Combined with individual vulnerabilities related to age, gender, and minority status, these factors shape depression risk among older women. Predisposing vulnerabilities, unequal enabling resources, and heightened social and economic tensions jointly influence mental health outcomes in these societies.

## 5. Research Implications

The findings of this study raise several important questions for future research, particularly regarding the complex interactions between socio-economic, political, and health-related factors and the mental health of women aged 50 and older. Future studies could explore the extent to which gender-specific differences in depression are amplified or mitigated when additional psychological and social variables are considered. Investigating psychosocial factors such as social isolation, family burdens, and intergenerational relationships could provide a better understanding of the mechanisms that influence the mental health of older women.

Additionally, future research should focus on understanding the regional disparities in depression and contextualizing these differences within the political and social structures of each country. In this regard, examining not only the predictors analyzed in this study but also other contributing factors, such as societal stigma and the role of healthcare, could add important insights. A more in-depth analysis of data from countries with lower participation rates or shorter study durations might provide new perspectives on the effects of short-term crises and political upheaval on the mental health of older women.

Finally, it would be valuable to design longitudinal, qualitative studies to gain more insight into the long-term impact of depression and the role of prevention and early intervention. The interactions between mental and physical health, particularly in an ageing society, could be explored in greater depth to develop specific prevention and treatment approaches for older women.

## 6. Practical Implications

The findings of this study have far-reaching practical implications for the policy and societal design of mental health promotion strategies for older women. Given the significant associations between depression and socio-economic, political, and health factors, it is clear, that the mental health of older women cannot be addressed in isolation within the healthcare sector. An integrated, interdisciplinary approach is needed that considers both healthcare access and the broader social and economic determinants of mental health.

Policy makers should implement targeted measures to address gender inequality, social isolation, and inadequate access to healthcare services. Especially important are initiatives aimed at improving access to mental health services in rural or economically disadvantaged regions to ensure equitable care for all older women. Low-threshold services, such as telephone or online counselling, could play a key role in reducing barriers to help-seeking, particularly in countries with underdeveloped healthcare infrastructures.

Furthermore, promoting educational and employment opportunities for women aged 50 and older, as well as improving pension and social benefits, could help reduce financial burdens and social inequality, which were identified as risk factors for depression. In Eastern European countries, where women are more likely to face lower pensions and higher unemployment, such measures could contribute significantly to improving both their quality of life and mental health.

Additionally, political campaigns that raise awareness of mental health issues and reduce stigma, particularly in Southern and Eastern European countries, should be considered. Increased public education and the promotion of a positive attitude towards mental health could help improve help-seeking behaviours and enhance societal acceptance.

Finally, designing longitudinal, qualitative studies would provide valuable insights to the specific needs of older women. Integrated programs that combine mental health support with social assistance could make a significant difference, especially in countries facing high elderly poverty rates and healthcare gaps.

## 7. Conclusions

Depression severity among older women across Europe is shaped by regionally distinct structural and cultural factors. Statistically significant predictors were identified in nine of 15 countries during the 2004–2015 study period, with the strongest and most consistent associations found in Eastern Europe (Poland, Czechia, Estonia). Persistent structural inequalities—including gender disparities, occupational segregation, limited healthcare access, and systemic corruption—exert long-term cumulative effects across the life course, intensifying the burden of depression, particularly among the oldest and most vulnerable women. Southern Europe (Spain, Greece) is characterized by weaker healthcare infrastructures and greater exposure to economic and policy shocks, with overlapping risk factors in Spain and austerity-driven constraints in Greece influencing both perceived and evaluated mental health needs. In Central and Northern Europe (Netherlands, Switzerland), relatively strong healthcare systems and enabling resources mitigate risk, though societal inequalities, historical discrimination, and minority-related vulnerabilities still shape depression outcomes.

In countries such as Estonia and Spain, the consistent presence of multiple predictors across age groups suggests a potential causal relationship between structural determinants and depression severity. In contrast, Central/Northern and Southern European countries displayed clinically relevant EURO-D scores without corresponding statistical predictors, implying the presence of unmeasured or complex psychosocial influences. Factors such as mental health stigma, age discrimination, and traditional gender roles may contribute to depression risk but are difficult to capture quantitatively within standard predictive models.

Notably, several predictors reflect subjective perceptions rather than objective conditions. For example, Switzerland exhibits low actual crime rates, yet the Global Peace Index suggests a heightened perceived sense of insecurity. Similarly, Spain’s corruption predictor highlights a discrepancy between public perception and OECD benchmarks. These findings underscore the psychological impact of perceived environmental threats, irrespective of objective data.

The treatment of minority groups also emerged as a significant societal factor. In Poland, Spain, and Switzerland, more inclusive attitudes—particularly toward LGBTQ+ individuals—were associated with lower severity of depression among women over 50. This suggests that social acceptance, equality, and cohesion may act as protective factors for mental health in ageing populations.

Overall, the findings underscore the importance of shared societal responsibility, in addition to individual risk factors, in addressing depression among older women. Policy development and intervention design should account for country-specific social, cultural, and historical contexts, with particular attention to vulnerable subgroups—most notably women over 80 years of age. Future research should further investigate potential causal links between structural predictors and mental health outcomes, with particular attention to variables such as tolerance, social cohesion, age-related discrimination, and protection of minority rights. Doing so may help develop more targeted, cost-efficient, and culturally appropriate prevention strategies for mental health in later life.

## Figures and Tables

**Table 1:** Predictors and corresponding plausibility criteria used to evaluate correlations with EURO-D scores. All variables are calculated regardless of age and sex unless otherwise stated.

Main Category	Variable	Source	Description	Frequency (Start Year)	Scale
Predictor Health	HAQ	IHME	Preventable mortality, risk-standardized death rates, mortality-to-incidence ratio	Ann. (1990)	0 to 100 (excellent)
	Psy/I	OECD	Practicing psychiatrists per 1000 inhabitants	Ann. (1991)	Absolute number
	GDP_H_	OECD	Healthcare expenditure (private/public hospitals, pharma, prevention)	Ann. (1970)	%GDP
Plausibility Criteria Health	OoP	WHO	Out-of-pocket health expenses (direct payments, non-reimbursed, in-kind)	Ann. (2000)	% share of current health expenditure
	Suicide Rate	World Bank	Female suicide rate per 100,000	Ann. (2000)	Quotient
	Life expectancy	World Bank	Female life expectancy at birth	Ann. (1960)	Years
Predictor Education, Employment and Finance	EI	UN	Ratio of actual to expected years of schooling (age 18)	Ann. (1990)	0 to 1 (highest attainable level of education)
	UR	OECD	Female unemployment (>1 yr) as % of total population	Quarterly (1963)	% share of population
	PPS_Pension_	SHARE	Only women over 50 years of age: ${PPS}_{Pension}=\frac{Pension}{{PPP}_{Country}}$	Waves 1–2, 4–6 (04–15)	Absolute value in €
Plausibility Criteria Education, Employment and Finance	Higher education for 50+women	UNECE	Tertiary Education (ISCED 5–8)	Ann. (2005)	% share of women
	STEM	World Bank	Female share of STEM graduates	Ann. (1998)	% share of women
	Old-age poverty	Eurostat	Women 65+, income <60% median (equivalized)	Ann. (2015)	% share of women
	IP-Index	Stanford [34]	Gender distribution across professions vs. ideal balance Gender distribution across	2007	0 to 1 (complete horizontal segregation)
Predictors Equality and Inclusion	GII	UN	Reproductive health (maternal mortality, adolescent births); empowerment (women in parliament, ≥secondary ed.); economic participation (female vs. male labour force rate)	Ann (1990)	0–1 (lack of gender parity)
	GI	UN	Population share; mean and cumulative income	Ann. (1963)	0–1 (inequality)
	REI	ILGA	Equality and non-discrimination (25 items); family (11); hate crimes/speech (8); legal recognition/physical integrity (13); civil society (6); asylum (6)	Ann. (2009)	% (the lower the less recognition of rights)
Plausibility Criteria Equality and Inclusion	GGGI	World Economic Forum	Economic participation and opportunity (labour force participation, wage equality, earned income, women in senior/technical roles); educational attainment (literacy, school enrollment); health and survival (sex ratio, healthy life expectancy); political empowerment (parliament, ministerial roles, years with female head of state)	Ann. (2006)	0 to 1 (full gender parity)
	GWG	Eurostat	Gender wage gap: % difference in gross hourly earnings (enterprises >10 employees)	Ann. (2006)	%-share
	GPG	Eurostat	Gender pension gap: % difference in pension income (statutory, occupational, private) for ≥65 y	(2010), ann. (2015)	%-share
	GPG-Index	Eurostat	Structural employment and pension inequalities: employment gap (employment histories, part-time rates, sectoral distribution, contract types); pension compensation (child-rearing credits, redistribution, supplementary pensions access)	2013	Composite score
Predictor Internal Security	GPI	Institute for Economics and Peace	National/international conflicts; national security; militarization	Ann. (2007)	1 to 5 (conflict-prone)
	CCI	World Bank	Political stability, corruption control, economic transparency, absence of violence, legal adherence, regulatory quality	Ann. (1996)	The lower the more corruption
	IQ	OECD	Immigrant share of total population	Ann (1995)	% share
Plausibility Criteria Internal Security	Robberies/ 100,000	The Global Economy	Property crimes involving force; excludes pickpocketing/extortion	Ann. (2009)	Absolute number
	CPI	Transparency International	Corruption facets: bribery, embezzlement, abuse of office, nepotism, state capture, integrity, judicial prosecution, bureaucracy, transparency laws, whistleblower protection	Ann (1995)	0–100 (high corruption)
	Interpersonal Violence	OECD	Women 15–49; self-reported violence, attitudes, legal frameworks	Sporadically (2000)	% share of female population in the same age group

**Table 2:** Number of study participants by wave (1, 2, 4, 5, 6) and age group.

	50 to 59 Years	60 to 69 Years	70 to 79 Years	80 to 89 Years	Σ
**Wave 1**	2.162	1.727	1.095	259	**5.243**
**Wave 2**	2.600	2.170	1.266	287	**6.323**
**Wave 4**	3.860	4.119	2.748	964	**11.691**
**Wave 5**	4.283	5.085	3.453	1.365	**14.186**
**Wave 6**	2.252	3.823	2.788	1.120	**9.983**
**Σ**	**15.157**	**16.924**	**11.350**	**3.995**	**47.426**

**Table 3:** Statistically significant predictors ranked in descending order by adjusted Pearson and the explanatory power of predictors I to V.

	Health			Education, Employment and Finance			Equality, Inclusion			Internal Security			Age Group	Adjusted Pearson Correlation Coefficient (r_adj_)	EURO D MV, 95%-CI, SD	Participating Years
	HAQ	Psy/I	GDP_H_	EI	UR	PPS_Pension_	GII	GI	REI	GPI	CCI	IQ				
PL PCC	II 0.26						I 0.27		IV 0.22	I 0.27	III 0.25	V 0.2	80 to 89 years	0.81	5 [4.65, 5.35] (SD = 0.18)	4
CZ PCC			I 0.26	II 0.15	I 0.26		I 0.26	II 0.15					80 to 89 years	0.72	3.6 [3.26, 3.94] (SD = 0.17)	8
EE PCC	I 0.18	I 0.18			I 0.18	II 0.16		III 0.14			II 0.16	I 0.18	80 to 89 years	0.67	4.1 [3.76, 3.94] (SD = 0.17)	4
PCC	I 0.1	I 0.1			I 0.1			I 0.1			I 0.1	I 0.1	70 to 79 years	0.46	3.5 [3.04, 3.96] (SD = 0.23)	
PCC	I 0.1	I 0.1			I 0.1			I 0.1			I 0.1	I 0.1	60 bis 69 years	0.46	3 [2.72, 3.28] (SD = 0.14)	
ES PCC	I 0.1				I 0.1				I 0.1	I 0.1	I 0.1		70 to 79 years	0.65	4 [3.18, 4.62] (SD = 0.36)	11
PCC	I 0.1	II 0.09			II 0.09			II 0.09	I 0.1	I 0.1	I 0.1		60 to 69 years	0.65	3.1 [2.54, 3.66] (SD = 0.28)	
NL PCC								III 0.1		I 0.1		II 0.1	80 to 89 years	0.58	2.6 [2.38, 2.82] (SD = 0.11)	9
CH PCC	II 0.19								I 0.2	III 0.19			80 to 89 years	0.54	2.5 [2.19, 2.81] (SD = 0.16)	11
SE PCC		I 0.1		II 0.09								I 0.1	80 to 89 years	0.54	2.9 [2.7, 3.1] (SD = 0.1)	11
SI PCC				I 0.14									80 to 89 years	0.35	3.2 [2.65, 3.75] (SD = 0.28)	4
GR PCC		I 0.1											80 to 89 years	0.32	3.5 [3.2, 3.8] (SD = 0.15)	3

MV = Mean Value, SD = Standard Deviation, CI = 95% Confidence Interval PCC = Pearson Correlation Coefficient in absolute values rounded to two decimal places.

## Data Availability

The datasets used and/or analyzed during the current study are available from the corresponding author on reasonable request. The data used in this study are from the Survey of Health, Ageing and Retirement in Europe (SHARE) and are available to registered researchers upon application through the SHARE Research Data Center. Restrictions apply to the public sharing of individual-level data due to ethical and legal considerations.

## Abbreviations

AT	Austria
BE	Belgium
CCI	Control of Corruption Index
CH	Switzerland
CZ	Republic of Czechia
DE	Denmark
EE	Estonia
EHIS	European Health Interview Survey
EI	Educational Index
ELSA	English Longitudinal Study of Ageing
ES	Spain
ESS	European Social Survey
ESEMeD	European Study of the Epidemiology of Mental Disorders
EU	European Union
EURO-D	European Depression Scale
FR	France
GDP	Gross Domestic Product
GDP_H_	Health expenditure in % of the Gross Domestic Product
GE	Germany
GI	Gini Coefficient (measure of inequality in assets)
GII	Gender Inequality Index
GPI	Global Peace Index
GR	Greece
HAQ-Index	Healthcare Access and Quality Index
ICT	Information and Communication Technology
IHME	Institute for Health Metrics and Evaluation
ILGA-Europe	International Lesbian, Gay, Bisexual, Trans and Intersex Association-Europe
IR	Immigration Ratio
IP-Index	Index of Occupational Segregation by Gender
IT	Italy
LASA	Longitudinal Aging Study Amsterdam
LGBTQ+	Lesbian, Gay, Bisexual, Transgender, Queer/Questioning, and other diverse sexual orientations and gender identities
MV	Mean Value
NHS	National Health Service (UK)
NL	Netherlands
OECD	Organisation for Economic Co-operation and Development
PPP	Purchasing Power Parity
PPP_Country_	Country-specific Purchasing Power Parity
PPS_Pension_	Pension Purchasing Power Standard
PL	Poland
REI	Rainbow Europe Index
SE	Sweden
SHARE	Survey of Health, Ageing and Retirement in Europe
SI	Slovenia
STEM	Science, Technology, Engineering, and Mathematics
WHO	World Health Organization
UN	United Nations
UNECE	United Nations Economic Commission for Europe
UR	Unemployment Rate
€	Euro
